# District mental healthcare plans for five low- and middle-income countries: commonalities, variations and evidence gaps

**DOI:** 10.1192/bjp.bp.114.153767

**Published:** 2016-01

**Authors:** Charlotte Hanlon, Abebaw Fekadu, Mark Jordans, Fred Kigozi, Inge Petersen, Rahul Shidhaye, Simone Honikman, Crick Lund, Martin Prince, Shoba Raja, Graham Thornicroft, Mark Tomlinson, Vikram Patel

**Affiliations:** **Charlotte Hanlon**, MRCPsych, PhD, College of Health Sciences, School of Medicine, Department of Psychiatry, Addis Ababa University, Addis Ababa, Ethiopia and Centre for Global Mental Health, Health Services and Population Research Department, Institute of Psychiatry, Psychology and Neuroscience, King's College London, UK; **Abebaw Fekadu**, MD, PhD, MRCPsych, College of Health Sciences, School of Medicine, Department of Psychiatry, Addis Ababa University, Addis Ababa, Ethiopia; **Mark Jordans**, PhD, HealthNet TPO, Research and Development Department, Amsterdam, The Netherlands and Centre for Global Mental Health, Health Services and Population Research Department, Institute of Psychiatry, Psychology and Neuroscience, King's College London, London, UK; **Fred Kigozi**, MBChB, MMed, Butabika National Referral and Teaching Mental Hospital, Makerere University, Kampala, Uganda; **Inge Petersen**, PhD, School of Applied Human Sciences, Howard College, University of KwaZulu-Natal, Durban, South Africa; **Rahul Shidhaye**, MD, MHS, Public Health Foundation of India, New Delhi, India and CAPHRI School for Public Health and Primary Care, Maastricht University, The Netherlands; **Simone Honikman**, MBChB, MPhil, Perinatal Mental Health Project, Alan J Flisher Centre for Public Mental Health, Department of Psychiatry and Mental Health, University of Cape Town, Cape Town, South Africa; **Crick Lund**, BA, BSocSci, MA, MSocSci, PhD, Alan J Flisher Centre for Public Mental Health, Department of Psychiatry and Mental Health, University of Cape Town, Cape Town, South Africa, and Centre for Global Mental Health, Institute of Psychiatry, Psychology and Neuroscience, King's College London, UK; **Martin Prince**, MD, MSc, MRCPsych, Centre for Global Mental Health, Health Services and Population Research Department, Institute of Psychiatry, Psychology and Neuroscience, King's College London, London, UK; **Shoba Raja**, BA, MA, BasicNeeds, Banasawadi, Bangalore, India; **Graham Thornicroft**, FRCPsych, PhD, Health Services and Population Research Department, Institute of Psychiatry, Psychology and Neuroscience, King's College London, London, UK; **Mark Tomlinson**, BA, BA(Hons), MA, PhD, Centre for Public Mental Health, Department of Psychology, Stellenbosch University and Department of Psychiatry and Mental Health, University of Cape Town, Cape Town, South Africa; **Vikram Patel**, MRCPsych, PhD, FMedSci, Public Health Foundation of India, Bhopal, Madhya Pradesh, India, Centre for Global Mental Health, London School of Hygiene and Tropical Medicine, London, UK and Sangath, Goa, India

## Abstract

**Background**

Little is known about the service and system interventions required for successful integration of mental healthcare into primary care across diverse low- and middle-income countries (LMIC).

**Aims**

To examine the commonalities, variations and evidence gaps in district-level mental healthcare plans (MHCPs) developed in Ethiopia, India, Nepal, Uganda and South Africa for the PRogramme for Improving Mental health carE (PRIME).

**Method**

A comparative analysis of MHCP components and human resource requirements.

**Results**

A core set of MHCP goals was seen across all countries. The MHCPs components to achieve those goals varied, with most similarity in countries within the same resource bracket (low income v. middle income). Human resources for advanced psychosocial interventions were only available in the existing health service in the best-resourced PRIME country.

**Conclusions**

Application of a standardised methodological approach to MHCP across five LMIC allowed identification of core and site-specific interventions needed for implementation.

Integrating mental healthcare into primary healthcare is recommended as the central strategy for addressing the large unmet need for effective and appropriate mental healthcare in low- and middle-income countries (LMIC).^1^ Integrated mental healthcare has the potential to be more holistic, less stigmatising and to promote human rights.^2^ Rigorous syntheses of the evidence base have defined the range of possible biopsychosocial interventions and delivery strategies for prioritised mental, neurological and substance use disorders in primary care in LMIC.^3–6^ However, in order to move from theoretically possible packages of interventions to actual implementation requires: (a) tailoring intervention packages to the opportunities and constraints available in the particular setting and (b) broadening consideration beyond the level of the health facility to interventions at the levels of the health service, system and community.

The PRogramme for Improving Mental health carE (PRIME)^7^ aims to implement, scale up and evaluate a coordinated district-level mental healthcare plan (MHCP) to integrate care for mental, neurological and substance use disorders into primary and maternal healthcare in five LMIC (Ethiopia, India, Nepal, South Africa and Uganda). This paper describes the contents of the district-level MHCPs developed in the five PRIME countries, emphasising: the similarities, as these are the core components that are generalisable across health systems; the variations, and what contextual factors may have influenced these; the human resource mix for implementation; the evidence gaps and how these are being addressed by the generation of new evidence; and the potential limitations of the resulting MHCPs. As far as we are aware, this is the first time that the same methodological approach has been applied to mental healthcare planning across five diverse LMIC.

## Method

### Settings

The PRIME countries include a low-income fragile state (Nepal), two low-income countries (Ethiopia and Uganda), one lower-middle-income country (India) and one upper-middle-income country (South Africa). In each country, an administrative healthcare unit, in this paper referred to as a ‘district’, was selected for the development and implementation of a comprehensive MHCP. Districts were largely considered to be representative of the country situation, although in Nepal the selected district was relatively better resourced than other districts in the country because of feasibility concerns. The characteristics of the districts have been described in detail.^8^ In brief, the population size of the selected district varied from 161 952 in Ethiopia to 1 311 008 in India. Most districts were predominantly rural (73% to 97%) apart from in South Africa (10%). Literacy ranged from 22% (Ethiopia) up to 88% (South Africa). Baseline mental healthcare provision in primary care was very low across all sites, with the activity of primary healthcare workers mainly limited to referral of people with severe mental illness who presented to the facility. Specialist mental health professionals were absent in the Ethiopia district and scarce in all other districts. Organisational systems to support mental healthcare within primary healthcare were absent or rudimentary.

### The MHCP

The purpose of developing an MHCP was to provide a detailed framework for implementation of mental healthcare integrated into primary care that could be used for subsequent scale up of mental healthcare within country, and also to serve as a template for similar settings in other LMIC. Each MHCP was initially structured into three levels: health service organisation, health facility and community. Each level of the MHCP was populated with packages of interventions considered to be necessary to provide comprehensive biopsychosocial care for people with priority mental, neurological and substance use disorders. The starting point for the intervention packages at the primary healthcare facility level was the evidence-based guidelines of the World Health Organization's (WHO's) Mental Health Gap Action Programme (mhGAP) intervention guide.^9^

### Formative work to develop an MHCP

The objectives of the formative phase of PRIME were to define which of the range of available evidence-based intervention packages were acceptable and feasible for delivery in a particular setting and to identify any other interventions required to ensure a comprehensive, feasible and sustainable MHCP. The development of a district-level MHCP was an iterative process, with various phases of formative work feeding into modification and refinement of the plan. The process was guided by the Medical Research Council framework for development and evaluation of complex interventions.^10^ Similar methodologies for the formative work were employed across the countries and are summarised in Table 1.

**Table 1:** Overview of formative study methods used by the PRogramme for Improving Mental health carE (PRIME) countries to develop a district-level mental healthcare plan

Activities	Nepal	Ethiopia	Uganda	India	South Africa
Prioritisation exercise	Yes^12^	Yes	Yes	Yes	Yes

Situation analysis	Yes	Yes	Yes	Yes	Yes

Consultation					
District community advisory board and country advisory groups	Yes	Yes	No	Yes	Yes

Theory of change workshops	4	2	2	2	3

Qualitative study					
Health service organisation Health facility/healthcare providers Community, people with mental, neurological and substance use disorders and caregivers	5 in-depth interviews 15 in-depth interviews 4 focus-group discussions (*n* = 36) 13 in-depth interviews 5 focus-group discussions (*n* = 48)	4 in-depth interviews 3 focus-group discussions (*n* = 18) 10 in-depth interviews 3 focus-group discussions (*n* = 26)	6 in-depth interviews 7 in-depth interviews 3 focus-group discussions (*n* = 21) 11 focus-group discussions (*n* = 63)	4 in-depth interviews 7 in-depth interviews 2 focus-group discussions (*n* = 16) 2 focus-group discussions (*n* = 16)	7 in-depth interviews 25 in-depth interviews 4 focus-group discussions (*n* = 22) 79 in-depth interviews

Costing exercise	Yes	Yes	Yes	Yes	Yes

Pilot study^11-15^	Implemented in 2 health facilities Routine monitoring data (*n* = 135 patients), -evaluation questionnaire (*n* = 77 patients, 32 of whom dropped out of care) and service providers (*n* = 11)	Implemented in 1 health facility Number of patients seen in facility post- training Health worker feedback on implementation	Implemented in 12 health facilities Evaluation of self- reported competence of health workers (*n* = 17) 4 in-depth interviews with health workers and 1 focus group discussion Health management information system case detection/ recording pre- and post-training	Implemented in 1 health facility Number of patients seen in facility post- training Monitored referrals to specialist clinics Assessed functioning of health management information system Qualitative study of acceptability and feasibility (*n* = 30)	Implemented in 1 health facility Number of patients seen in facility post-training and pathways through care Qualitative process evaluation 10 in-depth interviews with care providers 12 interviews with patients 4 in-depth interviews with caregivers

The formative work did not proceed in a linear manner, with several elements proceeding in tandem and the order varying by country. Each element of the formative work will now be described.

#### Working from the evidence base

The starting point for development of district MHCPs was the existing evidence base and experience base for integrating mental healthcare into primary healthcare.^4,6,8,16,17^ Following the theory of change (ToC) workshops (see below), specific areas requiring more detailed evidence were identified and a series of literature summaries was produced for each of these areas.

#### Consultation

A community advisory board was established in each of the PRIME districts comprising a range of relevant stakeholders; for example, representatives from the district health administration, traditional and religious healers, caregivers/patients, police and teachers. In addition, a country management group was formed, composed of the research team and national representatives from the Ministry of Health and patient/caregiver organisations. Regular meetings with the community advisory board and country management group allowed consultation over the appropriateness of the evolving MHCP and were an important vehicle for garnering political support.

#### Prioritisation exercise

Although the mhGAP intervention guide includes evidence-based guidelines for ten prioritised mental, neurological and substance use disorders, for initial implementation a subgroup of the priority disorders was selected in each country. Prioritisation took account of national priorities, for example, as outlined in existing mental health policies and plans, but local district priorities took precedence. Details of the approach used for prioritisation of mental, neurological and substance use disorders are given elsewhere.^7,11–15,18^

#### Situation analysis

A baseline situation analysis was carried out to identify the specific challenges and opportunities for integrated mental healthcare within each of the PRIME district sites. A situation analysis tool was developed for the purpose (downloadable from www.prime.uct.ac.za/images/prime/PRIME_Final_Situational_analysis_Tool.pdf). The PRIME situation analysis tool relied mostly on information available in the public domain. Details of the methods employed for the situation analysis have been described.^8^

#### ToC workshops

ToC is a systematic and participatory approach to development of a complex intervention (such as an MHCP) that engages all relevant stakeholders and seeks to work backwards from the desired final outcome to identify all of the steps required to achieve that outcome.^19^ In the case of PRIME, the ultimate goal of the MHCP was to improve clinical, social and economic outcomes for people with priority mental, neurological and substance use disorders living within the PRIME districts. In each PRIME country, a series of ToC workshops was carried out, as detailed in Breuer et al.^20,21^ The resulting ToC maps identified the key goals to be achieved by the MHCP, the underlying assumptions and indicators for evaluating the success of implementation at every step along the way. The ToC maps were closely linked to the MHCP as each of the identified ToC goals needed to be linked to a package of interventions within the MHCP.

#### Qualitative study

A qualitative study was carried out in each of the country sites using in-depth interviews and focus-group discussions with key informants from each level of the proposed MHCP: health service organisation, health facility and community. Details of the qualitative methodology employed are available in this supplement,^11–15^ and the numbers of interviews and focus groups are summarised in Table 1. A framework approach to analysis was used.^22^ The main objectives of the qualitative study were to: (a) identify the essential components of the district-level MHCP, and (b) explore how these MHCP components could be implemented in practice to achieve culturally and contextually acceptable, feasible and sustainable mental healthcare for people with priority mental, neurological and substance use disorders.

#### Modelling the cost of the MHCP

A costing tool developed for implementation of mhGAP^23^ was customised to each country context and used to estimate costs of scenarios based on different assumptions, for example, with respect to types of interventions included and level of coverage anticipated. The outputs of the costing exercise were used to inform discussions with key stakeholders when deciding on feasible and scalable MHCP components.^24^

#### Cross-country information-sharing

Throughout the development of the country MHCPs there was extensive cross-fertilisation of ideas between countries and with the cross-country partners. The MHCPs were presented at the annual PRIME meetings in 2012 and 2013. In preparation for the 2013 meeting, each of the PRIME MHCPs was reviewed formally within the PRIME consortium and both plans and feedback discussed in-depth at the meeting. The monthly teleconferences involving country principal investigators and cross-country partners also provided opportunities for discussions around the MHCP. The PRIME email network was used to disseminate relevant publications to the country teams often generating email discussion across the consortium about the implications for the emerging MHCPs.

#### Pilot work

Each country carried out piloting of implementation of the MHCP with a view to further refining the MHCP. The methodologies employed in the pilot studies vary across countries and are outlined in Table 1. The pilot studies involved implementation in between 1 and 12 health facilities, with evaluation of the number of patients seen post-training of the primary healthcare facility health workers. Feedback on the new service was obtained from health workers and patients using quantitative (Nepal) and qualitative (Ethiopia, India, Uganda, South Africa) methods.

### Cross-country analysis of MHCPs

The country MHCPs were reviewed in-depth with emphasis on comparing the MHCPs arising in countries of different resource levels. The goals and planned interventions for each level of the MHCP in each country were summarised in tabular form. Patterns of similarity and difference were noted and tested back with the country teams. A similar process was followed to compare the cross-country human resource requirements of the MHCPs. Evidence gaps, plans for new evidence generation and residual limitations of the MHCPs were also reviewed and compared across the countries.

Ethical approval was obtained from the relevant research ethics committees within each country as well as from the institutions of cross-country partners. Informed, voluntary consent was obtained from participants in the qualitative and pilot studies.

## Results

### Overall MHCPs

**Fig. 1:**
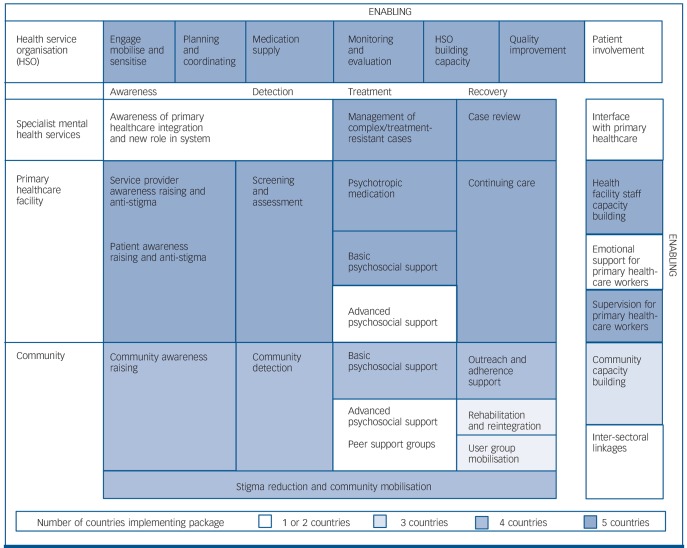
Shared and distinct intervention packages included in mental healthcare plans across the PRogramme for Improving Mental health carE (PRIME) countries.

The individual country MHCPs are described in detail in this supplement.^11–15^ A summary of the shared and distinct intervention packages included in MHCPs across the PRIME countries is given in Fig. 1. Enabling packages support the intervention packages at the health service organisation, facility and community levels.

Online Tables DS1–3 give details of the MHCP package components by country for the health service organisation, facility and community levels. Not all elements of the MHCP were considered to be feasible for implementation straight away, leading to plans for two phases of implementation in Nepal, Ethiopia and South Africa (‘immediate’ and ‘delayed’). Details of the human resources required to deliver each component of the MHCP packages across the PRIME countries are included in online Table DS4.

There were slightly different approaches between countries in their identified priority disorders. In the low-income PRIME countries (Ethiopia, Nepal and Uganda), epilepsy was included as an additional priority disorder in the MHCP (alongside psychosis, depression and alcohol use disorders), reflecting the existing health service structures in those countries whereby epilepsy is usually treated by mental health professionals. All countries except South Africa utilised the broad definition of psychosis employed within the mhGAP intervention guide, whereas South Africa only focused on schizophrenia. In Ethiopia and India, bipolar disorder was also selected as a priority. Following piloting, greater emphasis was given to medically unexplained somatic symptoms in the India MHCP. The choice of service within which to integrate mental healthcare was also affected by country context. In South Africa, the chronic disease service within primary healthcare was deemed the best target for integrating mental healthcare in response to country and policy priorities. In all other countries, where acute, communicable diseases still hold sway, the general primary healthcare setting was the target for integration.

### The MHCP at the health service organisation level

#### Commonalities and contextual variations

The goals of the MHCP at the level of health service organisation were shared across most of the PRIME countries with respect to engagement, advocacy, awareness raising and programme management (Table DS1 and Fig. 1). The need for ongoing meetings with district-level planners and coordination of integrated mental healthcare was included in all MHCPs and the establishment of a multisectoral community advisory board in all countries except Nepal and Uganda. The MHCPs in Uganda and South Africa are able to rely on routinely collected data on individuals seen in the health facilities because of the existence of functional health management information systems (HMISs), whereas in the other PRIME countries there is a need to institute parallel systems for obtaining this information. In Ethiopia and Nepal it is also necessary for PRIME to procure psychotropic medication for the initial implementation of the MHCP, reflecting the absence of medication in primary healthcare at baseline, with agreements in place that the district health administration will take over the role of ensuring a reliable medication supply as implementation proceeds. Only in South Africa was there an existing forum for multisectoral collaboration, whereas for the other PRIME countries the MHCP required establishment of new systems for multisectoral working.

#### Human resources

The mental health coordinator within the district health administration in Ethiopia was not a mental health specialist. In Ethiopia and South Africa, the health personnel being trained as trainers of primary healthcare staff in mhGAP guidelines were not mental health specialists. Ethiopia, Uganda and South Africa MHCPs included a ‘training of trainers’ model for building up district-level capacity to train primary healthcare facility-level workers, but in Ethiopia it was found to be necessary to rely on PRIME team expertise (from psychiatrists) for the initial phases of training. The India and Nepal MHCPs relied on training being delivered by the PRIME team mental health specialists (psychiatrists and psychologists).

At the health service organisation level in Nepal, the PRIME team will carry out most of the programme management activities to support MHCP implementation because of fragmentation of the existing health system administration in this post-conflict state. In Ethiopia and India, the MHCPs rely on PRIME providing support to administrators and coordinators within the existing systems, which are present but weak. In both Uganda and South Africa, coordination of mental healthcare will be largely carried out by the existing district health administration. In all countries except South Africa, quality improvement components of the MHCP were dependent on PRIME staff working together with the primary healthcare facility managers and district health administration.

### The MHCP at the facility level

#### Specialist mental health services

During the formative work, it became apparent that the district MHCPs needed to include an additional level outlining the role of specialist mental health professionals in relation to delivery of mental healthcare in primary healthcare (Table DS2). All of the country MHCPs, incorporated training of mental health specialists in their new role in the new service, except Uganda. The planned interface between primary healthcare and mental health services varied across countries, with specialist mental health clinics actually conducted in the subdistrict hospital linked to primary healthcare services in India, and variable levels of involvement in supervision and consultation across the other PRIME countries.

The availability of specialist mental health workers varied substantially across the PRIME country districts, with none available within the Ethiopian district. In the low-income countries (Ethiopia, Nepal and Uganda), it was necessary to employ mental health specialists through PRIME to carry out supervision of primary healthcare workers.

#### Primary healthcare facility

**Commonalities and variations**. See Table DS2. In South Africa, an existing set of intervention guidelines designed for the chronic disease service (Primary Care 101 (PC101)),^25^ was adapted to be consistent with the mhGAP intervention guide.^9^ In all other countries the mhGAP intervention guide was used to train primary healthcare facility-based health workers.

Only South Africa included facility-based ‘advanced psychosocial interventions’ (defined as ‘interventions that take more than a few hours of a healthcare provider's time to learn and typically more than a few hours to implement’^9^) in the first phase of MHCP implementation. In Nepal, primary healthcare workers were trained in brief psychosocial interventions that were focused on specific symptoms and referred people requiring advanced psychosocial interventions to a community-based counsellor. Emotional support interventions for primary healthcare workers were only included in the MHCP in South Africa.

**Human resources**. In both middle-income country districts (South Africa and India), confirmatory diagnosis for people with psychosis was made by a mental health specialist or doctor before being referred back to primary care for ongoing treatment. In contrast, in the low-income countries, only acutely disturbed individuals or ones with complex psychosis were referred for medical or specialist review and, for the majority of individuals, the diagnosis was made by non-specialised mid-level primary healthcare workers. Initiation of non-emergency medical treatment was restricted to medical doctors in South Africa and to mental health specialists in India but permitted by mid-level primary healthcare workers in the other countries.

Facility-based advanced psychosocial interventions using existing cadres of health workers were only feasible in South Africa and required employment of non-health sector staff in Ethiopia.

### The MHCP at the community level

#### Commonalities and variations

Community interventions aiming to increase mental health awareness, generate demand for mental healthcare, improve detection and reduce stigma were strongly emphasised within the MHCPs (Table DS3), but in South Africa such community-level interventions were not feasible in the initial phase of implementation. This was because of the need to synchronise with government plans for training of community health workers that are yet to commence. The MHCPs at community level all incorporated outreach mechanisms for patients who are lost to follow-up at the facility. Intervention packages to promote rehabilitation and reintegration were planned in three of the PRIME countries (Ethiopia, Uganda and South Africa). All countries planned interventions to mobilise patients for advocacy, although in three of the PRIME countries (Ethiopia, India and Nepal) this was not part of the initial implementation. Peer support groups were planned in all PRIME countries except Ethiopia, but were only part of the initial implementation in two countries (Uganda and South Africa). Community-based advanced psychosocial interventions were only included in the Nepal MHCP.

#### Human resources

In all PRIME countries, except Uganda, it was possible for the MHCP to make use of salaried community health workers. In Uganda the MHCP relied on health volunteers. Community-based rehabilitation and reintegration interventions were dependent on a non-governmental organisation (NGO) in Uganda (Basic-Needs)^26^ and in South Africa auxiliary social workers from an NGO (the North West Mental Health Society) and health promoters at the clinics. A new cadre of community-based rehabilitation worker was introduced in Ethiopia. Similarly, the advanced psychosocial interventions in the Nepal MHCP were to be delivered by a new cadre of ‘community counsellor’ employed by an NGO (Transcultural Psychosocial Organization Nepal).

The availability of expertise in the area of psychosocial interventions influenced development of the MHCPs in the PRIME sites, with greater capacity to deliver advanced psychosocial interventions present in the PRIME teams led by clinical psychologists (in Nepal and South Africa).

### Evidence gaps and innovations

See online Fig. DS1 for a summary of evidence generation activities to support implementation of the PRIME MHCPs. The need for better evidence to inform aspects of the MHCPs was recognised across the PRIME countries. Plans for new evidence generation in the PRIME district sites are targeted mostly at the enabling intervention packages, the development of advanced psychosocial interventions and interventions at the community levels of the MHCP. In terms of the enabling components of the MHCP, the following have been developed and will be evaluated: pilot schemes for patient/caregiver involvement in service improvement and quality improvement (Ethiopia) and HMIS indicators for integrated mental health (Ethiopia) as part of the PRIME-linked Emerald Project (www.emerald-project.eu); orientation of facility managers to integrated chronic care (South Africa); supportive supervision for primary healthcare workers delivering mental healthcare (Ethiopia, South Africa); measures of competence of primary healthcare workers trained in psychosocial interventions (Nepal, Ethiopia, South Africa); and an intervention to promote primary healthcare worker well-being (Ethiopia). Advanced psychosocial interventions will be developed and evaluated for the following mental, neurological and substance use disorders: depression in general primary healthcare attendees (Nepal, Ethiopia); depression in chronic care patients (South Africa); maternal depression (Ethiopia) and alcohol use disorders (Nepal, Ethiopia, South Africa). Evaluations of newly developed community-based rehabilitation in Ethiopia and psychosocial rehabilitation groups in South Africa for people with schizophrenia will be conducted. A newly developed tool for proactive community identification of people with mental, neurological and substance use disorders and evaluation of an intervention to enhance community outreach and adherence support for people receiving long-term mental healthcare will be conducted in Nepal. An investigation of potential areas for collaboration with traditional and religious healers will be carried out in Ethiopia and development of service models for integration of mental healthcare into primary healthcare-based maternal and child healthcare is underway in South Africa.

## Discussion

The MHCPs developed for implementation across five diverse LMIC as part of the PRIME project outline the intervention packages that aim to achieve improved clinical and functional outcomes in people with priority mental, neurological and substance use disorders living within a district. The district MHCPs spanned four levels: health service organisation, specialist mental health services, primary care facilities and the community, and incorporated a range of evidence-based biopsychosocial interventions and system-level ‘enabling’ packages. There were strong cross-country similarities in the goals of the MHCPs across the different levels. The intervention packages required to achieve these goals were more variable across the countries. The human resource mix for MHCP implementation tended to show a gradient across country income levels with greater reliance on more highly qualified and specialist health workers in the middle-income countries. The limitations of the existing evidence base for implementation have stimulated plans for generation of new evidence, with particular focus on community interventions, socioculturally contextualised advanced psychosocial interventions and health system interventions.

### System-level planning

In each of the countries, MHCP components within the health service organisation level and community level were as prominent as facility-based interventions, underlining the importance of these other levels of the health system for effective integration of mental healthcare into the primary care service. In addition to the mhGAP intervention guide,^9^ which is largely concerned with delivery of evidence-based treatment packages in health facilities, the WHO is developing training resources for the system-level interventions required for a coordinated service. However, the existing evidence base in this area is weak. This, coupled with an historical lack of emphasis on the system-levels interventions required for implementation, is likely to have been an important factor contributing to the lack of sustainability of previous efforts to integrate mental health into primary care in LMIC.^17^ In the PRIME-linked Emerald Project, the distinctive approaches to mental health system strengthening being employed across the PRIME sites will be evaluated, particularly with respect to quality improvement, operationalising and adapting chronic disease models of care, patient involvement and developing appropriate indicators of successful integration of mental healthcare.

The impact of community awareness-raising interventions on increasing demand for locally available mental healthcare has been demonstrated in an area of Nigeria with low baseline levels of awareness about mental, neurological and substance use disorders and their treatability.^27^ In the context of a research study in Ethiopia, community key informants trained using vignettes of typical case presentations were able to identify psychosis with high sensitivity^28^ and a more structured approach to community case identification is being evaluated in the PRIME Nepal district site.^12^ All of the PRIME district MHCPs included a component related to outreach and adherence support for people with mental, neurological and substance use disorders requiring ongoing care, making use of community health workers and volunteers who interface with primary care services. Community health workers have tended to be seen as something of a panacea to all community ills, with the result that they may become overloaded trying to respond to the immense unmet need for healthcare in most LMIC.^29^ Combined with the low priority given to mental health within the public health agenda of most LMIC, maintaining mental health outreach activities of this cadre of health worker may be especially challenging. Reliance on community volunteers as part of a delivery system for mental healthcare has also been found to be problematic in the past because of difficulty with sustainability.^26^

### Putting the ‘psychosocial’ into biopsychosocial

An important critique of the global mental health movement that drives the PRIME initiative is the alleged imposition of a biomedical world-view of interventions.^30^ Although mhGAP recognises the social determinants of mental disorder and emphasises social interventions for all of the priority mental, neurological and substance use disorders, the starting point for intervention is facility-based delivery of care by health workers within an illness model. In this regard, a strength of the PRIME MHCPs is the inclusion of community-based interventions to promote a more broadly conceptualised recovery, in terms of social and economic functioning as well as to bring about improvement in clinical symptoms. Concerns remain, however, about the feasibility of such an approach in practice, with the risk that a more narrowly biomedical MHCP focused on prescription of medication will be easier to implement and that social issues might be pushed aside. In Ethiopia, India and Uganda, the absence of existing contextually appropriate advanced psychosocial interventions and the means for their delivery meant that these were not included in the phase one implementation plan. This area is particularly important for new evidence generation, recognised in recent priority-setting exercises for global mental health research.^31^

The human rights of people with mental, neurological and substance use disorders were given high priority within the MHCPs, with particular emphasis on the right to health and access to mental healthcare. The implicit assumption was that treatment of the mental, neurological and substance use disorders would be the best intervention against human right violations such as restraint.^32^ Interventions to counter stigma and discrimination against people with these disorders in community life were also present in all of the MHCPs. Legislative frameworks safeguarding the rights of people with these disorders when receiving mental healthcare were absent in all of the PRIME countries except South Africa. In such contexts the MHCP interventions to strengthen patient advocacy and mobilisation are of vital importance to help to strengthen checks and balances within the system.

### Tailoring MHCPs to the country situation

Variation in the baseline readiness of the PRIME districts to integrate mental healthcare into primary care was identified by country-specific situation analyses.^8^ Our cross-country analysis of the MHCPs developed for each PRIME district underscores this variation in terms of the intervention packages included in each MHCP and the timing of implementation of different packages. Our findings support the proposition of a core district-level MHCP suitable for the most poorly resourced settings that can evolve and expand as and when further resources become available. In this core MHCP, external system and organisational support are likely to be necessary at start-up, particularly in fragile state settings where health systems are often fragmented and weak, coupled with augmented support from specialist mental health professionals to ensure the quality of training and supervision. A strong reliance on mid-level facility-based health workers to diagnose, assess, prescribe and providing basic psychosocial care and continuing care for people with mental, neurological and substance use disorders would be at the heart of the core MHCP, but would be ineffective without accompanying community-level awareness raising to generate demand for services. Rehabilitation and reintegration would need to harness existing community resources.

In better-resourced LMIC, a district MHCP might be expected to require less external technical support at start-up and on an ongoing basis. Integration of MHCP programme management into existing systems will be more feasible. As more highly trained human resources become available, more complex skills, such as initial diagnosis and management of new presentations of psychosis, may be carried out by doctors and specialist mental health workers rather than mid-level health workers. A broader range of interventions, including advanced psychosocial interventions, may be feasible.

### Limitations

The MHCPs presented in this paper focus on services within the public sector and by not-for-profit organisations, despite recognition of the potential contribution of the private sector in LMIC.^33^ Although sustainability was one consideration for inclusion of interventions within the MHCP, several of the MHCPs draw on new cadres of worker and established systems that will be parallel to existing public sector services. This approach is justified by the low levels of existing investment in mental healthcare and the need to establish proof of concept for certain types of intervention that cannot be supported within the existing system, such as advanced psychosocial interventions; however, the extent to which these interventions will be sustainable after international funding is discontinued is unknown.^34^

In this paper we report on the district-level MHCPs developed through application of a standardised, participatory and mixed quantitative–qualitative methodological approach across districts in five diverse LMIC. Such an approach has not been utilised previously and has both strengthened our knowledge of how to develop district MHCPs and highlighted the ways in which context informs what is possible in a particular setting. The PRIME district MHCPs will now be implemented and subjected to a rigorous and comprehensive evaluation.^35^
